# Evaluation of a redesigned two-day trauma course incorporating blended learning compared with the traditional three-day trauma course: a retrospective non-inferiority study

**DOI:** 10.1186/s13017-026-00714-x

**Published:** 2026-07-14

**Authors:** Marly Iskander, Frederike J. C. Haverkamp, Leo M. G. Geeraedts, Sef J. A. van Bilsen, Edward C. T. H. Tan

**Affiliations:** 1 Department of Surgery, Radboud University Medical Center, Geert Grooteplein-Zuid 10, 6525 GA Nijmegen, The Netherlands; 2 Department of Surgery, Section Trauma Surgery, Amsterdam University Medical Center, Amsterdam, The Netherlands; 3 Department of Anesthesiology, Elisabeth-TweeSteden Hospital, Tilburg, The Netherlands

**Keywords:** Multidisciplinary trauma course, Self-assessed confidence, Blended learning, Traditional learning, Technical skills, Non-technical skills

## Abstract

**Background:**

During the multidisciplinary Definitive Surgical and Anesthetic Trauma Care (DSATC) course, trauma specialists are trained to apply damage control principles to polytrauma patients. The course was redesigned in 2020 from a three-day, traditional format to a restructured two-day format combining online and traditional learning methods to enhance course quality and learning outcomes. Considering that learning opportunities on the work floor are scarce, continuous course evaluation is crucial to ensure benefits for medical professionals and their patients.

**Methods:**

This retrospective non-inferiority study compared self-assessed confidence in technical and non-technical skills between a cohort in the traditional DSATC course and a cohort in the redesigned DSATC course. Self-assessed confidence ratings were collected through quantitative pre-course and post-course questionnaires. Mean changes in confidence ratings were calculated and compared using independent samples t-tests with a non-inferiority margin of -0.5. Sub-analyses investigated differences between participants of the same profession, between physicians with different levels of experience, and the influence of individual preparation on self-assessed confidence in skills before on-site training.

**Results:**

A total of 180 (21%) participants completed the pre-course and one-day post-course questionnaires completely. Both cohorts showed increased self-assessed confidence in skills following course completion, with the redesigned DSATC cohort demonstrating non-inferior improvements compared with the traditional cohort in general technical skills (mean difference (MD) 0.07, lower bound one-sided 97.5% confidence interval (CI) -0.14, *p* < 0.001 for non-inferiority) and in general non-technical skills (MD 0.09, lower bound one-sided 97.5% CI -0.09, *p* < 0.001 for non-inferiority). Self-assessed confidence in non-technical skills declined in the 2024 redesigned course group following individual preparation (MD -0.21, *p* = 0.042).

**Conclusions:**

The redesigned format of the DSATC course is not inferior to the traditional model in improving self-assessed confidence in technical and non-technical skills. Exploratory findings suggest that individual pre-course preparation may be associated with greater awareness of learning needs and support self-directed learning. These results indicate that a traditional, one-size-fits-all approach to training might not fully address differences in learners’ needs. Blended learning could support individualized learning pathways. Further research should investigate the effectiveness of such pathways in multidisciplinary trauma education and their relation to competence, clinical performance and patient outcomes.

**Supplementary Information:**

The online version contains supplementary material available at https://doi.org/10.1186/s13017-026-00714-x.

## Introduction

The effective treatment of critically injured patients requires an integrated set of technical and non-technical skills, such as acute surgical skills, communication, and teamwork [1–3]. Educational opportunities aimed at the treatment of polytrauma patients are crucial to improve the readiness and competence of medical professionals in trauma care, since this influences the quality of care provided to patients [4, 5]. This is particularly important for high-acuity, low-occurrence (HALO) events, which are life-threatening injuries or procedures that are infrequently encountered in clinical practice, yet demand rapid and effective management when they occur [6]. However, learning opportunities in the clinical work environment are often limited by factors such as increased nonoperative management and the unpredictable and time-sensitive nature of trauma cases, which complicate the planning and delivery of educational initiatives, resulting in a lack of on-the-job learning opportunities [3, 7, 8].

In recognition of the need for trauma care education, various courses have been developed. Examples include Advanced Trauma Operative Management (ATOM), Advanced Trauma Life Support (ATLS), Advanced Surgical Skills for Exposure in Trauma (ASSET), Definitive Surgical Trauma Care (DSTC) and Definitive Anesthetic Trauma Care (DATC) courses [9–12]. Participation in such courses has the potential to enhance self-assessed confidence, clinical performance, knowledge, and the efficiency of team-based patient care [1, 12–14].

The Dutch DSTC and DATC courses were the first to be combined into one multidisciplinary course: the Definitive Surgical and Anesthetic Trauma Care (DSATC) course. The Dutch DSATC course has been in place since 2009 and provides learning opportunities to national and international participants involved in trauma care, such as intensivists, scrub nurses, anesthesia nurses, surgeons and anesthesiologists [11, 15, 16]. The intention is to equip participants with knowledge and skills regarding the application of damage control principles to polytrauma patients through interactive workshops, group discussions, and lectures [15, 16]. Hands-on experience with trauma cases is offered through simulation in a regulated environment, enabling skill development while eliminating risks for individual patients [6, 11, 16, 17]. Before participation, participants are required to complete ATLS or European Trauma Course (ETC) training and to be at least in the final year of residency, as the DSATC course is an advanced trauma care course focusing on damage control principles and multidisciplinary trauma management. The unique multidisciplinary approach, which distinguishes the DSATC course, improves teamwork and team-based care for severely injured patients [1, 11, 14, 18].

In 2020, the traditional three-day on-site DSATC course was redesigned into a two-day format incorporating blended learning, alongside changes to course duration, preparation and workshop structure (Appendix A). The redesigned course combines online modules with two days of in-person training while maintaining the same amount of hands-on experience. A full DSATC course typically includes 36 surgical participants, 12 anesthesiologists, 12 anesthesia nurses and 12 scrub nurses. During training sessions, supervision is provided by surgical faculty members in a 1:4 instructor-to-participant ratio to three surgical participants and one scrub nurse, and by anesthesiologic faculty members in a 1:2 instructor-to-participant ratio to one anesthesiologic participant and one anesthesia nurse. This allows participants to actively participate and assist in damage control procedures. Preparation for the previous course consisted mainly of theoretical study through course manuals, whereas the current format provides preparatory material through the renewed DSATC manual [19], a webinar and an online learning environment, comprising pre-recorded lectures, e-learnings and virtual simulation. This change, prompted by the COVID-19 pandemic and guided by previous faculty and participant feedback, can potentially enhance learning by offering greater flexibility for individual learners’ needs, reinforcing theoretical knowledge, and improving preparation for practical training. Online learning complements traditional methods but cannot replace hands-on learning [20, 21]. Given the significance of educational quality for participant development and, consequently, its potential implications for patient outcomes, it is essential to evaluate how differences in course design influence educational quality and learners’ self-assessed confidence [14].

Therefore, the objective of this study is to compare self-assessed confidence in skills between participants who attended a previous traditional DSATC course and those who completed the new redesigned DSATC course. With this approach, the study aims to generate actionable learning points applicable to the DSATC and similar courses. To our knowledge, few studies have examined both technical and non-technical skills within such multidisciplinary settings. Previous studies also highlighted the need for research on the multidisciplinary aspects of trauma care courses. These points have been identified as gaps in research [1, 18, 22], which this study seeks to address.

## Methods

### Study design and setting

This retrospective non-inferiority cohort study included two cohorts of attendees from the DSATC course at the Radboud University Medical Centre (Radboudumc) in Nijmegen, the Netherlands. Cohort 1 consisted of participants from traditional DSATC courses between 2016 and 2019. Cohort 2 consisted of participants who took part in the redesigned DSATC course between 2021 and 2024.

## Participants

The study population comprised all participants (*n* = 843) in the DSATC course at Radboudumc between 2016 and 2024. The participants included were mainly attending and resident surgeons and anesthesiologists, scrub nurses and anesthesia nurses from various countries.

Course participants were informed about the conduct of this study throughout the DSATC courses and were invited to complete the corresponding questionnaires. These questionnaires were solely distributed and used for research purposes and therefore did not preclude participation or certification. Participation was voluntary and consent to participate in this study was implied by the completion of the questionnaires. This study was determined to be exempt from the Dutch Medical Research Involving Human Subjects Act (WMO) by a Medical Ethics Committee.

## Data collection

All course participants received an invitational email to anonymously fill out quantitative digital questionnaires at five instances: one day before the course, and one day, three months, one year, and two years after the course. Reminders were sent four weeks later. Participants from the 2024 redesigned DSATC course group received an additional questionnaire before receiving access to the individual preparatory materials. Answers were automatically collected and organized into secure data files on Radboudumc servers by the Department of Surgery. Given the voluntary nature of the questionnaires, participants who wished not to participate could opt to be unsubscribed from future invitations and withdraw at any time.

The questionnaires were identical at each timepoint (Appendix B) and data from the questionnaires were analyzed to reveal actionable learning points for the DSATC and similar courses. The questions encompassed participant characteristics, including sex, age, and profession, as well as technical skills (e.g. trauma laparotomy, treating head trauma) and non-technical skills (communication, teamwork, leadership). Participants assessed their confidence in each skill using a five-point Likert scale (1 not confident – 5 very confident). Skills questions concerned general skills that applied to all participants, as well as specific skills for each of the main professions, namely surgeons (including orthopedic surgeons), anesthesiologists and anesthesia/scrub nurses. The questionnaires were based on previous resources consisting of course objectives and content, expert opinions, and a similar questionnaire utilized in a study by Gaarder et al. [23]. Previous studies have utilized and published on the questionnaire [11, 16]. Minor differences existed between the traditional and redesigned course questionnaires, mainly regarding the newly developed individual preparations. The previous questionnaire was published in the 2020 study by Tan et al. [11].

### Data analysis

Participant characteristics and mean evaluation grades, collected from annual digital course evaluations, were analyzed descriptively. These are presented as the mean ± standard deviation (SD) or as numbers with percentages. Participant characteristics were analyzed comparatively between the traditional and redesigned course cohorts via a chi square test for categorical data and an independent samples t-test for numerical data. Prominent participant feedback from the evaluations and overall self-assessed skills progression observed throughout the surveys were also reported descriptively.

The primary outcome measures were the mean changes in self-assessed confidence in general technical and non-technical skills, compared between the cohorts. For the comparative analysis, self-assessed confidence ratings of general skills questions were combined to compute the primary outcome variables: confidence in general technical and non-technical skills. Mean confidence ratings were calculated from the pre-course and one-day post-course questionnaires for each cohort. The data had been previously collected.

Additional sub-analyses were conducted. One sub-analysis compared the mean change in self-assessed confidence in skills between the cohorts for each of the main professions: surgeons (including orthopedic surgeons), anesthesiologists and anesthesia/scrub nurses. The outcome variables were computed from specific skills questions from the pre-course and one-day post-course questionnaires. Surgeons’ non-technical skills covered leadership and teamwork, while nurses’ and anesthesiologists’ non-technical skills exclusively covered teamwork. A second sub-analysis compared the mean change in self-assessed confidence from pre-course to one-day post-course between attending and resident physicians within each cohort. A final sub-analysis was conducted using data from the 2024 DSATC course group (*n* = 49), to examine how individual preparations might have influenced self-assessed confidence in general technical and non-technical skills before the on-site training.

**Fig. 1:**
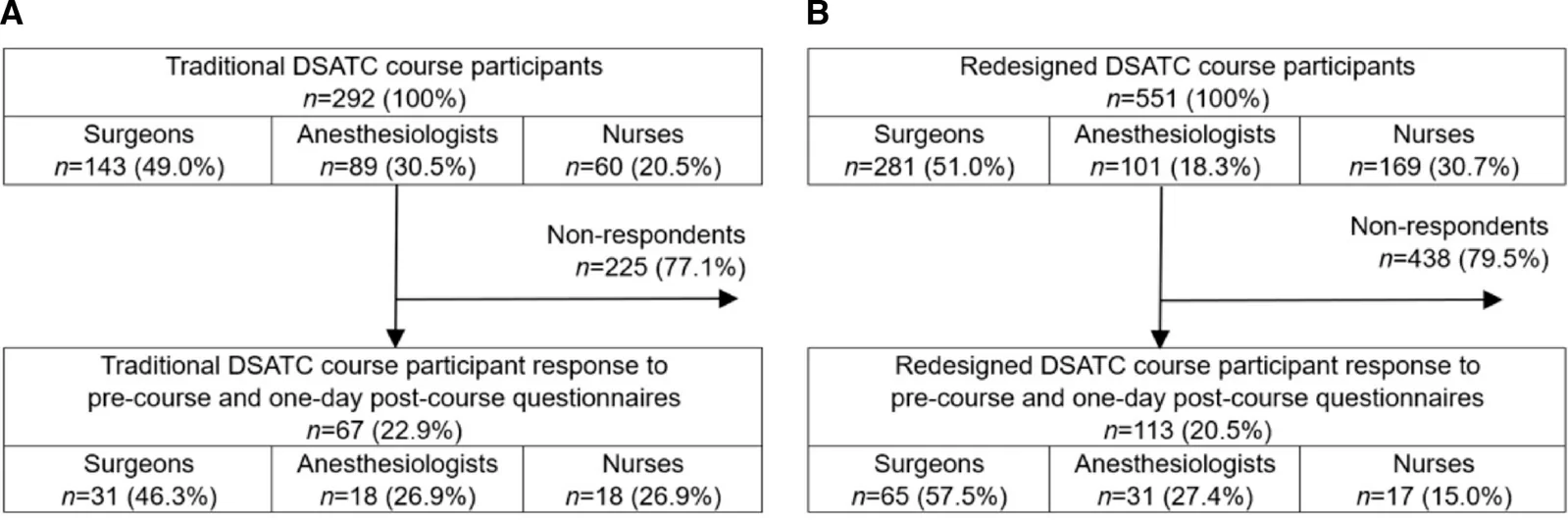
**A** Cohort 1 participant and respondent distribution* n* number of respondents. Percentages are rounded may therefore not sum to 100.0%. Surgeons include orthopedic surgeons.** B** Cohort 2 participant and respondent distribution* n* number of respondents. Percentages are rounded may therefore not sum to 100.0%. Surgeons include orthopedic surgeons.

Participants who completed both the pre-course and one-day post-course questionnaires were included in the statistical analysis. Those who did not complete both or only completed them partly were excluded (Fig. 1). The questionnaires beginning three-months post-course were not analyzed statistically because of low response rates. Furthermore, participants of whom it was unclear which questionnaire they intended to complete were excluded, and missing answers were excluded per analysis.

All inferential statistics were conducted using IBM SPSS Statistics (version 29). An independent samples t-test was deemed the appropriate statistical method for all analyses except the final sub-analysis, for which a paired samples t-test was used. For the analysis of participant characteristics and for the sub-analyses, a two-sided α ≤ 0.05 was used.

For the primary analysis, non-inferiority was assessed using a 97.5% confidence interval (CI), consistent with a one-sided α ≤ 0.025 commonly applied in non-inferiority analyses [24]. Accordingly, interpretation focused on the lower bounds of the corresponding one-sided 97.5% CI. A pre-defined non-inferiority margin of -0.5 points on the five-point Likert scale was used. The null hypothesis stated that the mean difference in change in self-assessed confidence between the redesigned and traditional cohorts was less than or equal to the non-inferiority margin: *H*_*0*_: *µ*_*redesigned DSATC*_ *- µ*_*traditional DSATC*_ *≤ -0.5*. The alternative hypothesis was *H*_*A*_: *µ*_*redesigned DSATC*_ *- µ*_*traditional DSATC*_ *> -0.5*. The non-inferiority margin was selected in consultation with a statistician at Radboudumc and was considered the largest difference that would not represent an educationally meaningful reduction in confidence, consistent with recommendations that non-inferiority margins should reflect the maximum acceptable loss of effect [24]. Given that each Likert category reflects a distinct level of self-assessed confidence, a difference smaller than half a scale point was considered unlikely to represent a practically relevant difference between course formats. This margin was also informed by the variability observed in previous findings [11, 16], with reported standard deviations of approximately 0.5–0.6 in one study [16].

## Results

### Participation and response rates

An overview of the study population before and after the application of the inclusion criteria is depicted in (Fig. 1). Participants of whom it was unclear which questionnaire they intended to complete were labeled non-respondents in the figure (*n* = 4). Among the 843 DSATC course participants between 2016 and 2024, 180 (21%) participants completed both the pre-course and one-day post-course questionnaires. The number of participants who completed each subsequent questionnaire declined, with 85 (10%) responses up to three-months post-course, 31 (4%) responses up to one-year post-course, and 9 (1%) responses up to two-years post-course. In total, 667 (79%) individuals completed at least one questionnaire. A comprehensive respondent distribution can be consulted in Appendix C.

## Descriptive analysis

Table 1 presents the background characteristics of the respondents who completed both the pre-course and one-day post-course questionnaires. There was significant heterogeneity between the cohorts. Compared with the traditional course cohort, the redesigned course cohort included a greater number of male participants (*p* = 0.021) and had a different distribution of professional roles: the redesigned course cohort had a lower proportion of nurses (*n* = 15% vs. *n* = 27%) and a greater number of orthopedic surgeons (*n* = 31 vs. *n* = 8) (*p* = 0.048). In addition, the redesigned course cohort comprised a greater proportion of resident physicians (*n* = 35, 31%) than the traditional course cohort (*n* = 9, 18%) (*p* = 0.025).

**Table 1:** Background characteristics of respondents

Characteristic	Traditional DSATC course	Redesigned DSATC course	Total
	*n* = 67	*n* = 113	*n* = 180
*Sex (n*,* %)* *			
Male	36 (54%)	80 (71%)	116 (64%)
Female	31 (46%)	33 (29%)	64 (36%)
*Age in years (mean*,* SD)*	40.3 (9.1)	40.3 (7.9)	40.3 (8.3)
*Profession (n*,* %)* *			
Surgeon	23 (34%)	34 (30%)	57 (32%)
Nurse	18 (27%)	17 (15%)	35 (19%)
Anesthesiologist	18 (27%)	31 (27%)	49 (27%)
Orthopedic surgeon	8 (12%)	31 (27%)	39 (22%)
*Seniority*^a^* (n*,* %)* *			
Attending physician	40 (82%)	61 (64%)	101 (70%)
Resident physician	9 (18%)	35 (36%)	44 (30%)
*Years of work experience*^b^* (mean*,* SD)*	15.2 (8.3)	16.1 (7.1)	15.8 (7.5)
*Hospital of employment (n*,* %)*			
Academic or military	28 (42%)	44 (39%)	72 (40%)
Peripheral or teaching	33 (49%)	67 (59%)	100 (56%)
Other	3 (4%)	0 (0%)	3 (2%)
Missing	3 (4%)	2 (2%)	5 (3%)
*Employed at a level 1 trauma center (n*,* %*)			
Yes	29 (43%)	63 (56%)	92 (51%)
No	5 (7%)	49 (43%)	54 (30%)
Missing	33 (49%)	1 (1%)	34 (19%)

*SD* standard deviation, *n* number of respondents. Percentages are rounded and may therefore not sum to 100.0%. ^a^ Only applicable to surgeons, orthopedic surgeons and anesthesiologists; percentages are expressed as a proportion of the total number of physicians in the corresponding cohort. ^b^ Only applicable to anesthesia nurses and scrub nurses. * Statistical analysis on the variable demonstrated significant differences between the cohorts.

**Fig. 2:**
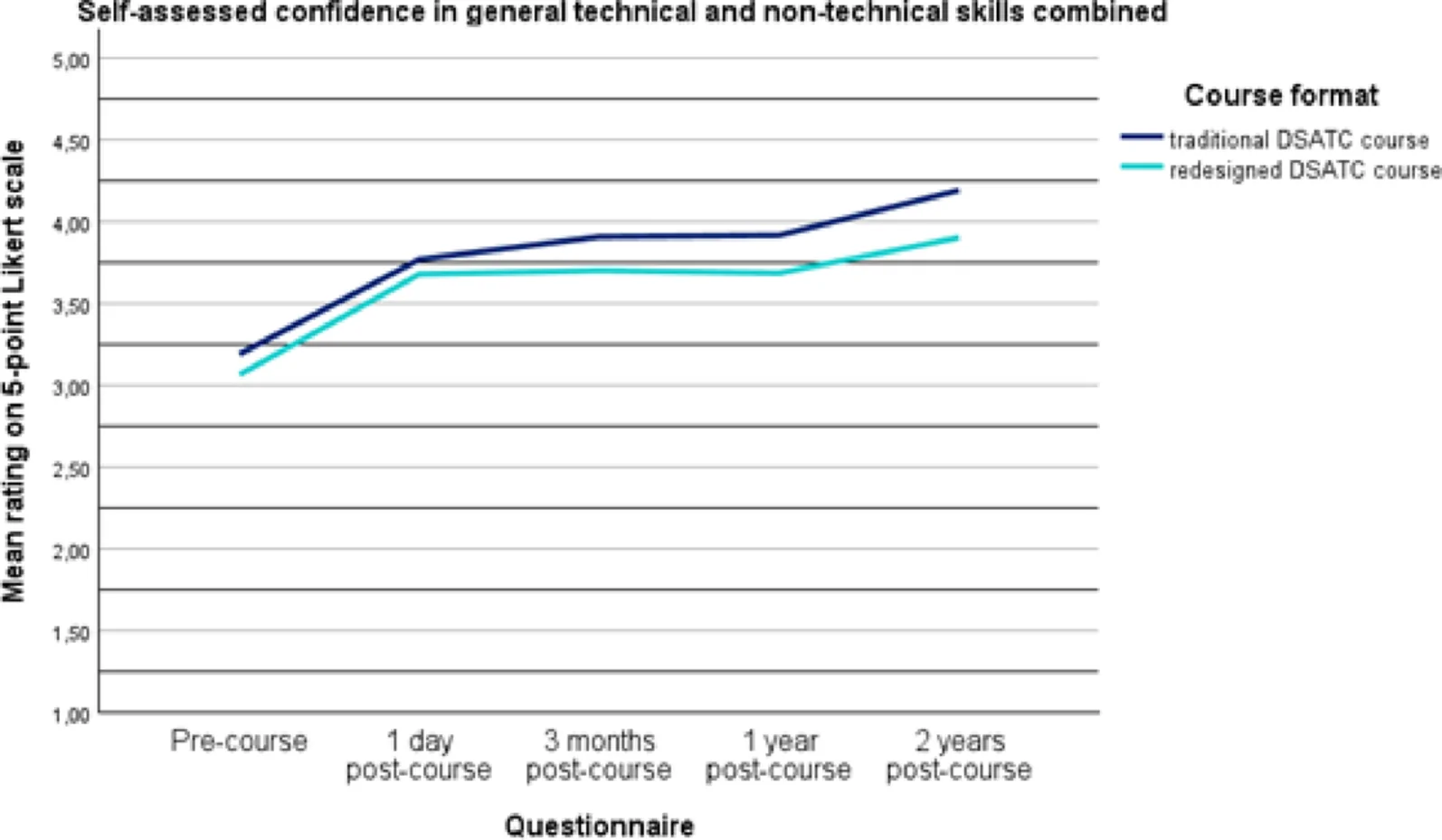
Longitudinal self-assessed confidence in general skills in the traditional versus redesigned DSATC course

Figure 2 depicts the longitudinal effect of both course formats on participants’ self-assessed confidence in skills. Compared with the redesigned DSATC cohort, the traditional DSATC cohort consistently demonstrated higher confidence levels, but the mean self-assessed confidence levels remained higher than the pre-course confidence levels throughout the follow-up period in both cohorts. However, this was not analyzed statistically.

Additionally, participants in the redesigned course cohort were asked to rate their level of agreement with the statement *‘individual preparation is not necessary prior to the course’* on a five-point Likert scale (1 completely disagree – 5 completely agree). The respondents demonstrated a mean change in agreement of -0.04 (SD 1.1) toward greater disagreement with the statement after completing the course compared to before the course (*p* = 0.002). The mean preparation time in this cohort was 14.2 hours (SD 7.9) for the online preparation modules and 18.3 hours (SD 11.8) in total, including course manuals and additional preparation.

## Primary analysis

For the primary analysis, the mean changes in self-assessed confidence in general technical and non-technical skills were compared between the traditional learning DSATC group (*n* = 67) and the redesigned DSATC group (*n* = 113) (Table 2). For general technical skills, the mean difference (MD) between the groups was 0.07, with a greater increase in self-assessed confidence in the redesigned DSATC group. The lower bound of the one-sided 97.5% CI was − 0.14 (*p* < 0.001 for non-inferiority). For general non-technical skills, the MD between the groups was 0.09, again with a greater increase in self-assessed confidence in the redesigned DSATC group. The lower bound of the one-sided 97.5% CI for this difference was − 0.09 (*p* < 0.001 for non-inferiority). For both skill categories, the lower confidence bound did not exceed the pre-specified non-inferiority margin of -0.5.

**Table 2:** Self-assessed confidence in general skills compared between two DSATC course formats

General skills^c^	Traditional DSATC *n* = 67			Redesigned DSATC *n* = 113			Mean difference (Lower bound 97.5% CI)^e^	*p* value^f^
	Mean rating (SD)^d^		Mean change (SE)	Mean rating (SD)^d^		Mean change (SE)		
	Pre- course	One-day post-course		Pre-course	One-day post-course			
*Non-technical*								
Communication	3.9 (0.6)	4.1 (0.6)	0.25 (0.08)	3.8 (0.7)	4.1 (0.6)	0.36 (0.06)	0.11 (-0.08)	< 0.001
Teamwork	3.6 (0.6)	4.1 (0.6)	0.51 (0.08)	3.5 (0.7)	4.0 (0.5)	0.52 (0.07)	0.01 (-0.20)	< 0.001
*Technical*								
Trauma laparotomy	3.3 (1.2)	4.2 (0.9)	0.94 (0.13)	2.5 (1.3)	3.7 (1.2)	1.19 (0.11)	0.26 (-0.08)	< 0.001
Damage control surgery	3.2 (1.2)	4.0 (0.9)	0.81 (0.12)	2.5 (1.2)	3.5 (1.2)	1.01 (0.10)	0.20 (-0.13)	< 0.001
Head trauma	3.0 (1.0)	3.6 (1.0)	0.55 (0.11)	2.8 (1.2)	3.2 (1.1)	0.42 (0.10)	− 0.13 (-0.43)	0.009
Neck trauma	2.9 (0.9)	3.6 (0.8)	0.73 (0.11)	2.7 (1.1)	3.4 (0.8)	0.70 (0.10)	− 0.03 (-0.34)	0.001
Blast injury	2.5 (0.9)	3.4 (0.9)	0.97 (0.10)	2.2 (1.0)	3.2 (1.0)	0.96 (0.09)	− 0.01 (-0.29)	< 0.001
Ballistic/penetrating injury	2.7 (0.8)	3.5 (0.9)	0.79 (0.09)	2.4 (1.1)	3.5 (1.0)	1.07 (0.10)	0.28 (0.02)	< 0.001
Burn injury	2.8 (0.8)	3.6 (0.8)	0.75 (0.09)	2.7 (1.0)	3.4 (1.0)	0.66 (0.08)	− 0.09 (-0.33)	< 0.001
*Pooled non-technical*	3.8 (0.5)	4.1 (0.6)	0.32 (0.07)	3.7 (0.6)	4.1 (0.5)	0.41 (0.05)	0.09 (-0.09)	< 0.001
*Pooled technical*	2.9 (0.7)	3.7 (0.7)	0.80 (0.07)	2.5 (0.9)	3.4 (0.8)	0.86 (0.07)	0.07 (-0.14)	< 0.001
*Combined*	3.4 (0.5)	3.9 (0.6)	0.56 (0.06)	3.1 (0.6)	3.8 (0.6)	0.64 (0.05)	0.08 (-0.07)	< 0.001

*SD* standard deviation, *SE* standard error of the mean, *n* number of respondents, *CI* confidence interval. ^c^ Absence of questions about teamwork, trauma laparotomy, damage control surgery and burn injury in the traditional course of 2019 reduced the number of respondents to *n* = 64 for these general skills. ^d^ Scale: 1 not confident – 5 very confident. ^e^ Mean difference between cohorts in the change in self-assessed confidence in skills from pre-course to one-day post-course, with negative values indicating a greater change in self-assessed confidence among traditional DSATC course participants compared to redesigned DSATC course participants. ^f^ Level of significance α ≤ 0.025 for non-inferiority (H_0_: µ_redesigned DSATC_ - µ_traditional DSATC_ ≤ -0.5).

### Sub-analysis

The first sub-analysis revealed no significant differences between the cohorts in self-assessed confidence in either specific technical or specific non-technical skills for each of the main professional groups.

The second sub-analysis examined differences in the mean change in self-assessed confidence between residents and attending physicians. In both cohorts, residents demonstrated a greater increase in self-assessed confidence in both technical and non-technical skills than attending physicians did, although the difference was not statistically significant. In the traditional DSATC group, the mean increase for residents exceeded that of attending physicians by 0.14 (*p* = 0.498) for technical skills and 0.28 (*p* = 0.175) for non-technical skills. In the redesigned DSATC group, the differences were 0.20 (*p* = 0.134) and 0.07 (*p* = 0.518) for technical and non-technical skills, respectively.

The final sub-analysis examined the impact of completing the new individual preparations on participants’ self-assessed confidence prior to the on-site sessions of the 2024 redesigned DSATC course. For both general technical and non-technical skills, self-assessed confidence declined after completing individual preparations. While the decline in confidence in technical skills was not significant (MD -0.06, *p* = 0.597), the decline in self-assessed confidence in non-technical skills was indeed significant (MD -0.21, *p* = 0.042).

### Course evaluations

The evaluation scores for the DSATC courses are summarized in Table 3. The 2017 traditional course received the lowest grade (mean 8.37 ± SD 1.46), whereas the 2024 redesigned course received the highest (mean 8.67 ± SD 0.74). Generally, the redesigned courses received higher grades than the traditional courses did.

**Table 3:** Annual DSATC course evaluation grades with response rates

	Course year							
	2016	2017	2018	2019	2021	2022	2023	2024
Grade (Mean, SD)^g^	8.50 (0.88)	8.37 (1.46)	8.49 (0.75)	8.41 (0.76)	8.56 (0.73)	8.63 (0.71)	8.50 (0.73)	8.67 (0.74)
Response rate (%, n/total)	96% (53/55)	67% (41/61)	92% (82/89)	91% (79/87)	64% (90/140)	54% (73/134)	72% (101/141)	63% (85/136)

*SD* standard deviation, *(n/total)* number of respondents/number of invitees. Percentages are rounded. ^g^ Scale: 1 very poor – 10 excellent.

Participants highlighted the importance of practical surgical skills and problem-solving across all evaluations. Despite this, participants from both course formats noted that the integration of crew resource management (CRM) principles into the individual preparation and on-site training remains underdeveloped. These principles cover non-technical skills such as problem-solving, leadership, situational awareness and decision-making [2]. While some found the anatomy and operative porcine laboratories morally challenging, they were considered indispensable by traditional course participants and very beneficial by redesigned course participants. Practical workshops were consistently rated as the best feature, whereas the quantity and quality of presentations were rated lower. Language barriers hindered the effective application of CRM principles.

Participant feedback on the individual preparation of the redesigned course revealed mixed views. While some found it useful and interactive, others, particularly nurses, indicated that it was too extensive, beyond their expertise and mostly aimed at surgeons. Non-technical skills preparation was seen as insufficient. Moreover, disparities in professional backgrounds affected perceptions of course relevance: nurses felt inadequately involved, anesthesiologists expressed a wish for more depth or cross-disciplinary insights, and orthopedic surgeons and lower-level trauma center participants reported feeling out of place. Despite this, practical workshops increased confidence in trauma management for some participants. Suggestions for course improvement differed by cohort, with traditional DSATC course participants favoring a reduction in on-site training by one day, whereas redesigned DSATC course participants favored less individual preparation or an additional on-site training day.

## Discussion

This study explored the impact of redesigning a multidisciplinary trauma care course from a three-day, traditional learning format to a two-day format incorporating blended learning on participants’ self-assessed confidence in technical and non-technical skills. The findings suggest that the redesigned format is not inferior to the traditional format in enhancing Dutch DSATC course participants’ self-assessed confidence, with both cohorts reporting increased confidence following course completion. Notably, participants in the redesigned cohort reported numerically greater gains in self-assessed confidence in non-technical skills and several technical skills.

Blended learning in healthcare education has been widely studied. Reported benefits include opportunities for personalized learning, flexibility in when and where to learn, enhanced knowledge acquisition, and a reinforced connection between theory and practice [20, 25]. In some cases, blended learning has been demonstrated as a non-inferior approach, such as in the study by Bergmans et al. (2023), in which a blended-learning approach was compared with traditional face-to-face teaching in the context of technical skill acquisition and retention in the ETC [26]. Even so, the variability in DSATC course participants’ feedback highlights how differences in course design, delivery, participant characteristics and individual engagement with blended-learning programs can lead to varied perceptions and experiences [25, 26]. This underscores the idea that online education – despite its benefits – cannot fully replace all aspects of in-person curricula and that both should be customized with the individual learner in mind [20, 21].

The individual preparations of the redesigned DSATC course further illustrate both the potential and challenges of blended learning. The cohort that completed the newly developed individual preparatory material reported lower baseline confidence in non-technical skills (i.e., prior to the on-site training sessions). Drawing on Kolb’s Experiential Learning Theory, the preparatory phase may facilitate reflective observation and abstract conceptualization through exposure to theoretical material without the benefit of hands-on experience [27]. This process may increase participants’ awareness of the complexity of the subject matter and of existing knowledge gaps, thereby influencing self-assessed confidence [28]. However, these processes were not directly assessed in this study and should therefore solely be interpreted as exploratory and hypothesis-generating.

Nevertheless, this phase may be a valuable component of the learning process, as it can encourage active and critical reflection on one’s understanding and may facilitate the recognition and formulation of learning goals [27]. For example, Smyth et al. (2012) reported that postgraduate students in a blended-learning program described developing an increased sense of responsibility for their own learning [29]. In support of this idea, participants in the redesigned DSATC cohort – despite the initial report of lower self-assessed confidence before the on-site training – ultimately reported similar and for several skills even numerically greater increases in self-assessed confidence after the course and retrospectively valued the preparatory component differently. In contrast, the traditional preparatory course manual was reported to be underutilized and may have had less influence on participants’ self-perceived confidence.

The redesigned Dutch DSATC course reduced on-site training from three days to two days, while introducing approximately one day of individual preparation. As a result, the overall educational time investment remained comparable, and the in-person component was restructured to maintain a similar amount of hands-on training within a shorter timeframe. Such changes in course duration and structure may be logistically favorable but could influence participants’ self-assessed confidence and learning experiences. Previous research by Chesov et al. (2026) suggested that short, targeted acute care courses can positively influence learner outcomes [30]. In this study, the World Health Organization International Committee of the Red Cross Basic Emergency Care (BEC) was delivered over the course of four days, whereas it is traditionally conducted as a five-day program [31]. Participants in the study by Chesov et al. valued the short course duration, although a desire for additional hands-on training opportunities was also expressed. Similar feedback was observed in this study, despite self-assessed confidence not showing inferiority compared with the traditional course format. The latter suggests that the redesigned educational approach may achieve similar confidence outcomes despite restructuring and shortening of the in-person component. However, the specific contribution of the individual redesign components cannot be isolated within the current study design.

Finally, some of the findings could be explained by differences between the cohorts. There was significant heterogeneity in terms of sex, profession and seniority. The redesigned cohort included more resident physicians than attending physicians. Although not statistically significant, resident physicians in both cohorts showed a tendency toward numerically larger increases in self-assessed confidence in both technical and non-technical skills. Differences in experience may influence self-assessed confidence, as less experienced participants may report lower baseline confidence but are just as likely to achieve similar levels of confidence as more experienced participants [32]. Additionally, the higher proportion of male participants in the redesigned cohort may have influenced self-assessed confidence levels, as prior studies suggest that self-assessments can vary by sex independently of measured performance, with women often underestimating their abilities relative to male colleagues [33]. Furthermore, written feedback revealed that differences in professional backgrounds between cohorts may have contributed to variation in how the course was experienced and valued, which in turn may have influenced the reported changes in self-assessed confidence. These factors should be considered when interpreting differences between the cohorts.

### Strengths and limitations

This study extends previous observations by investigating how course modifications affect participants’ self-assessed confidence in skills in a multidisciplinary trauma education setting. As recommended by other studies, all trauma team members [1, 18] and both technical and non-technical domains were included (Appendix B) [18, 22]. Additionally, the inclusion of attending physicians is noteworthy since they can also benefit from such courses due to the scarcity of on-the-job learning opportunities, but there is a paucity of literature reporting on attending physicians as trainees [22]. The diversity of participants in terms of profession and professional experience enhances the potential generalizability of the findings to other multidisciplinary trauma training contexts, particularly those aiming to integrate blended learning. However, the primary aim of this study was to compare the traditional and redesigned DSATC course formats rather than to compare the DSATC course with other advanced trauma courses. Direct comparisons with similar simulation-based trauma courses would additionally be complicated by substantial heterogeneity in educational design and means of assessment, study populations and outcome measures. Future research using more standardized assessment methods may help facilitate comparisons between advanced trauma education programs.

With 180 participants in the primary analysis, this study represents a large evaluation of trauma professionals’ self-assessed confidence in skills following a multidisciplinary trauma course. However, these participants represented 21% of the 843 course participants between 2016 and 2024, and response rates declined substantially in subsequent questionnaires (Appendix C). This introduces a risk of response bias in terms of motivation and learning experiences. The presence or effect of response bias could not be further identified due to absence of data on non-respondents. Correspondingly, comparisons between the cohorts were sometimes limited by missing answers (Tables 1 and 2).

In addition, although both course formats targeted comparable trauma care professionals within the same Dutch DSATC program, there was significant heterogeneity in participant characteristics between the cohorts, which, as discussed earlier, may have influenced study findings. No statistical adjustment was made for these differences because of the small subgroup sample sizes. The observational, non-randomized study design further limits the possibility of causal interpretation. Importantly, the subgroup analyses that were conducted in this study were exploratory in nature and should therefore be interpreted cautiously.

Moreover, the results were entirely based on self-assessments, which should be considered with some caution when interpreting them. Self-assessments are essential in adult educational processes to enhance learning but are subject to various factors that influence accuracy, such as work experience and self-perceived competence [33, 34]. They do not necessarily correspond to objective competence or clinical performance and are subject to bias. Objective assessments using procedure-specific checklists or global rating scales, or the use of video playback would be helpful to assess objective performance and skills [35].

Although the effectiveness of trauma care education on performance has been observed through, for example, increased confidence and teamwork [23, 36], it remains unknown exactly to what extent and precisely how increased confidence translates to improved performance in daily practice and, most importantly, improved patient outcomes [36, 37]. To our knowledge, few studies have proven the effectiveness of such training in reducing patient morbidity and mortality [5]. While improved patient outcomes are the main goal of trauma care courses such as the DSATC course, this study was not designed to evaluate such outcomes, which remain a challenging area to investigate because of the many environmental variables [38].

## Conclusion

This study provides insights into the potential implications of a redesigned model in multidisciplinary trauma education incorporating blended learning methods and a shorter duration of on-site training, as demonstrated by the Dutch DSATC course. Both the traditional and redesigned course cohorts showed increased self-assessed confidence following the course, with the redesigned format meeting criteria for non-inferiority to the traditional format in the short term. The quantitative data and participant feedback offer complementary insights into the implications of this approach, emphasizing the role of individual learner backgrounds and preferences in cultivating confidence in both technical and non-technical skills.

Given the importance of a multidisciplinary approach in preparing trauma teams for the treatment of critically injured patients, current educational methods may need further alignment with the needs of individuals from diverse professional backgrounds. Although improved confidence is a desirable outcome of training, it does not directly translate into improved competence. Future research should therefore further explore how educational design influences self-assessed confidence, while isolating the effects of individual components and incorporating objective measures of competence and performance.

## Supplementary Information

Below is the link to the electronic supplementary material.

Supplementary Material 1

Supplementary Material 2

Supplementary Material 3

## Data Availability

All data generated or analyzed during this study are included in the published article and its supplementary information files. The primary research data are not publicly available and are owned by the Department of Surgery of the Radboud University Medical Center (Radboudumc) in Nijmegen, the Netherlands.

## Abbreviations

HALO: High-acuity low-occurrence
ATOM: Advanced trauma operative management
ATLS: Advanced trauma life support
ASSET: Advanced surgical skills for exposure in trauma
DSTC: Definitive surgical trauma care
DATC: Definitive anesthetic trauma care
DSATC: Definitive surgical and anesthetic trauma care
ETC: European trauma course
Radboudumc: Radboud university medical center
WMO: Dutch medical research involving human subjects act
SD: Standard deviation
CI: Confidence interval
MD: Mean difference
CRM: Crew resource management
BEC: Basic emergency care
