# Supplementary material for: Evaluation of a redesigned two-day trauma course incorporating blended learning compared with the traditional three-day trauma course: a retrospective non-inferiority study
Source: World J Emerg Surg. 2026 Jul 14;21:57. doi: 10.1186/s13017-026-00714-x (PMC13679183; doi:10.1186/s13017-026-00714-x)
Supplement: Supplementary file 1 [file 13017_2026_714_MOESM1_ESM.docx]

**Appendix A: Comparison of the traditional and redesigned DSATC course formats**

**Table A1 Side-by-side timelines of the traditional and redesigned DSATC courses**

|  | Traditional DSATC course |  | Redesigned DSATC course |
| --- | --- | --- | --- |
| 1 | Course registration | 1a  1b | Course registration  Pre-preparation questionnaire (#0)^h^ |
| 2 | Start of preparation: provision of course manuals and instructional videos^i^ | 2  3 | Start of preparation: provision of literature and access to an online learning environment with various mandatory materials^i^  Webinar one month prior to on-site training |
|  |  | 4 | Continuation of preparation: proceeding with the literature and materials in the online learning environment^i^ |
| 3 | Pre-course questionnaire (#1) | 5 | Pre-course questionnaire (#1) |
| 4 | On-site training (3 days):  - Keynote lectures  - Topic presentations  - Plenary case discussions  - 5-station workshop carousel (*animal  laboratory, anatomy, fire side case  discussions, lectures, meet the expert*)  - Daily debriefing and feedback sessions | 6 | On-site training (2 days):  - Keynote lectures  - Interactive case discussions  - Fire side case discussions  - 3-station workshop carousel (*animal  laboratory, anatomy, meet the expert*)  - Daily debriefing, feedback sessions and  review of learning goals |
| 5 | One day post-course questionnaire (#2) | 7 | One day post-course questionnaire (#2) |
| 6 | Three months post-course questionnaire (#3) | 8 | Three months post-course questionnaire (#3) |
| 7 | One year post-course questionnaire (#4) | 9 | One year post-course questionnaire (#4) |
| 8 | Two years post-course questionnaire (#5) | 10 | Two years post-course questionnaire (#5) |

*DSATC* Definitive Surgical and Anesthetic Trauma Care
All questionnaires were administered solely for research purposes and not as a means of assessment ^h^ The pre-preparation questionnaire was first distributed to participants of the 2024 DSATC course ^i^ See table A2

**Table A2 side-by-side comparison of the individual preparation per profession for the traditional and redesigned DSATC course**

| Profession | Traditional DSATC preparation | Redesigned DSATC preparation^j^ |
| --- | --- | --- |
| Surgeon | Literature:  - DSTC manual  Video material:  - CD-ROM/SD card included in  the DSTC manual with videos  regarding surgical approaches  performed in the animal laboratory  - Penetrating abdominal trauma^k^ | Literature:  - DSATC manual by Kenneth D  Boffard (includes videos)  *-* Topknife by Hirschber & Mattox  Video lectures:  - Animal ethics  - Anatomy ethics  - Vascular and extremity trauma  - Dissection room dos and don’ts  E-learnings:  - Crew Resource Management  - Damage Control Surgery and  anesthesia  - Ballistics  - Blast  - Trauma of the neck  - Trauma of the chest – surgery  - Trauma of the abdomen  - Massive hemorrhage and  coagulation  Exam^l^:  - 50 questions  - 70% needed to pass  - Participation is still possible if  passing score is not obtained  - 3 attempts allowed  - Exam becomes visible only after  completing the mandatory modules  - Personal learning goals need to be  formulated and submitted after    Optional material:  - Modules for other professions  - Additional websites, articles and  books |
| Anesthesiologist | Literature:  - Anesthesia reader (collection of  articles with topics such as  the Injury Severity Score, Glasgow  Coma Scale and anatomic  profiles)  - Selected chapters from the DSTC  manual (general introduction,  Crew Resource Management,  pre-hospital aspects, resuscitation  physiology, transfusion in trauma,  damage control, organ system  injury: the neck, organ system  injury: the chest, organ system  injury: the pelvis, head trauma,  burns, special patient situations,  critical care of the trauma patient,  trauma anesthesia, physiology of  trauma) | Literature:  - DSATC manual by Kenneth D  Boffard (includes videos)  *-* Topknife by Hirschber & Mattox  Video lectures:  - Animal ethics  - Anatomy ethics  - Dissection room dos and don’ts  - Coagulation  E-learnings:  - Crew Resource Management  - Damage Control Surgery and  anesthesia  - Ballistics  - Blast  - Trauma of the neck  - Trauma of the chest – anesthesia  - Airway management in trauma  patients  - Massive hemorrhage and  coagulation  - Surgical procedures for  anesthesiologists  - Introduction to animal laboratory  specific physiology and anatomy of  pigs  Exam^l^:  - 50 questions  - 70% needed to pass  - Participation is still possible if  passing score is not obtained  - 3 attempts allowed  - Exam becomes visible only after  completing the mandatory modules  - Personal learning goals need to be  formulated and submitted after    Optional material:  - Modules for other professions  - Additional websites, articles and  books |
| OR nurse | Literature:  - OR nurse reader (collection of   articles with topics such as  transfusion protocols for massive  hemorrhage, Damage Control  Surgery and anatomy)  Video lectures:  - General videos on the DSATC  course | Virtual Reality:  - Animal lab case abdominal trauma  Video lectures:  - Animal ethics  - Anatomy ethics  - Dissection room dos and don’ts    E-learnings:  - Welcome OR nurses  - Crew Resource Management  - Damage Control Surgery and  anesthesia  - Trauma of the chest – surgery  - Trauma of the neck  - Trauma of the abdomen    Exam^l^:  - 21 questions  - 70% needed to pass  - Participation is still possible if  passing score is not obtained  - 3 attempts allowed  - Exam becomes visible only after  completing the mandatory modules  - Personal learning goals need to be  formulated and submitted after    Optional material:  - Modules for other professions  - Additional websites, articles and  books |
| Anesthesia nurse | No specific preparation material was distributed to anesthesia nurses. | Video lectures:  - Animal ethics  - Anatomy ethics  - Dissection room dos and don’ts  E-learnings:  - Crew Resource Management  - Damage Control Surgery and  anesthesia  - Trauma of the neck  - Trauma of the chest – anesthesia  - Airway management in trauma  patients  - Surgical procedures for  anesthesiologists  - Neurotrauma for anesthesia nurses  - Massive hemorrhage and  coagulation for anesthesia nurses  - Introduction to animal laboratory  specific physiology and anatomy of  pigs    Exam^l^:  - 20 questions  - 70% needed to pass  - Participation is still possible if  passing score is not obtained  - 3 attempts allowed  - Exam becomes visible only after  completing the mandatory modules  - Personal learning goals need to be  formulated and submitted after    Optional material:  - Modules for other professions  - Additional websites, articles and  books |

*DSATC* Definitive Surgical and Anesthetic Trauma Care, *DSTC* Definitive Surgical Trauma Care,
*OR* operating room
^j^ the individual preparation is estimated to take approximately 8-10 hours of work
^k^ this video module was offered exclusively to participants of the 2018 and 2019 DSATC courses and was available on <https://academy.incision.care/>
^l^ the preparatory exams were a component of the mandatory preparation intended to support engagement with the material and were not used for assessment purposes
