# Supplementary material for: Evaluation of a redesigned two-day trauma course incorporating blended learning compared with the traditional three-day trauma course: a retrospective non-inferiority study
Source: World J Emerg Surg. 2026 Jul 14;21:57. doi: 10.1186/s13017-026-00714-x (PMC13679183; doi:10.1186/s13017-026-00714-x)
Supplement: Supplementary file 2 [file 13017_2026_714_MOESM2_ESM.docx]

**Appendix B: Questionnaire of the redesigned DSATC course**

**Abbreviations**
I, II, III, IV	Section 1, 2, 3, 4 of the questionnaire
S	Question intended for surgeons including orthopedic surgeons
A	Question intended for anesthesiologists
N	Question intended for scrub nurses and anesthesia nurses

*Characteristics of participants*

I1 – What is your gender?
I2 – What is your age?
I3 – I am a	(surgeon/nurse/anesthesiologist)
I4 – Specialty
I5 – Type of hospital
I6 – I work in a level 1 trauma center
I7 – My experience in charge of trauma cases
I8 – The amount of years I worked with the following function at this moment: If a certain function does not apply in your situation, this should be noted as 0 years. Per example a third-year resident (AIOS) should be noted down as: resident (AIOS) 3 and attending (medisch specialist) 0.
I10 – Experience as a nurse
I12 – Momentum of when this questionnaire is filled in
I13 – When did you follow the course?

*Course preparation*
II1 – Have you read the DSATC course manual of Kenneth D Boffard?
II2 – Have you read the book Topknife?
II3 – Have you read additional articles recommended in the e-modules?
II4 – Have you read additional articles recommended in general?
II5 – Have you read additional articles that were not recommended?
II6 – Did you complete all the e-modules?
II8 – Did you watch the additional video lectures on blast, gunshot and trauma radiology?
II9 – How many hours did you spend on preparation on the website (www.dstc-datc.nl), the online DSTC or DATC platform and the Brightspace environment?
II10 – Could you give an estimate of how much time you spend on preparation for the course (excluding logistical matters such as travel and lodging)?

*Score each question from 1 up to 5. 1 - Completely false 2 - False 3 - Neutral 4 - True 5 - Completely true*

*Pre-course assessment*
III1 - Lectures play an important role in learning
III2 - The e-module 'Trauma of the chest' to the course was of added value
III3 - The e-module 'Trauma of the abdomen' to the course was of added value

III4 - The e-module 'Crew Resource Management (CRM)' to the course was of added value

III5 - The e-module 'Massive hemorrhage and coagulation/REBOA' to the course was of added value

III6 - The e-module 'Trauma of the neck and mediastinum' to the course was of added value

III7 - The e-module 'Airway management in trauma patients' to the course was of added value

III8 - Individual preparation is not necessary prior to the course

III9 - I prefer e-modules above lectures

III10 - I prefer e-modules above reading assignments

*How do you self-assess your confidence in the skills needed to treat a trauma patient? Confidence can be scored from 1 (not confident) up to 5 (confident).*

*Communication during surgical trauma emergency situations*
IVSAN1 – Timely informing team members about the situation
IVSAN2 - Ensuring team members receive and understand the information correctly
IVSAN3 - Timely asking information from team members
IVSAN4 - Sharing necessary information with team members to enable effective team functioning

*Teamwork during surgical trauma emergency situations*

IVS1 – Encouraging input concerning the situation from all members

IVS2 – Ensuring team members know what their role is during the situation

IVAN1 – Speaking up concerning the situation of the patient

IVAN2 – Speaking up about the roles of the members of the team

IVSAN5 – Timely organizing activities to ensure effective team functioning

IVSAN6 – Recognizing the needs of other team members

IVAN3 – Assessing the situation and thinking ahead about necessities

IVAN4 - Fulfilling specific tasks to achieve goals

*Leadership*

IVS3 – Assessing the injuries and prioritize

IVS4 – Delegating tasks of the team members to achieve goals

*Skills*
IVSN1 – Trauma laparotomy and packing

IVSN2 – Surgical approach of the abdomen; this includes: systemic abdominal exploration, medial visceral rotation left, medial visceral rotation right, trauma to the liver and packing, trauma to the spleen (including splenectomy), trauma to the pancreas, trauma to the duodenum and closure of the abdomen.

IVSAN7 – Damage control surgery

IVSN5 – Bleeding; this includes massive hemorrhage, coagulation in trauma and thrombosis profylaxis

IVSN7 – Extremes of age

IVSN8 – Abdominal compartment syndrome

IVSN14 – Urological trauma

IVSN15 – Surgical approach of the chest; this includes: opening the chest, median sternotomy, cardiac and lung repair, thoracic aorta isolation and clam shell.

IVSN16 – Extremity trauma

IVSN17 – Vascular trauma

IVSN18 – Pelvic trauma

IVSAN9 – Head trauma

IVSAN10 – Neck trauma

IVSAN11 – Thoracic trauma

IVSAN12 – Blast injury

IVSAN13 - Ballistic/penetrating injury

IVSAN14 - Burn injury (including chemical)

IVSAN15 - Intervention radiology in trauma

IVA1 – Damage control anesthesia

IVA2 – Managing shock

IVA3 – Managing massive blood transfusion

IVA4 – Intelligent fluid therapy

IVA5 – Managing coagulopathy after major trauma

IVA6 – Resuscitative phase (first 24 hours post-injury)

IVA7 – Early life support phase (24 to 72 hours post-injury)

IVA8 – Prolonged life support phase (after 72 hours post-injury)

IVA9 – Managing multiple organ dysfunction syndrome

IVA10 – Evaluation of metabolic disturbances

IVA11 – Recognition and treatment of head trauma

IVA12 – Managing a mass casualty situation

IVA13 – Pediatric resuscitation

IVA14 – Resuscitation of the elderly

IVA15 – Drowning

IVA16 – Burn evaluation and resuscitation

IVA17 – Thoracic injury and ventilatory strategies

IVA18 – Surgical airway

IVA19 – Trauma laparotomy
