# Supplementary material for: Evaluation of a redesigned two-day trauma course incorporating blended learning compared with the traditional three-day trauma course: a retrospective non-inferiority study
Source: World J Emerg Surg. 2026 Jul 14;21:57. doi: 10.1186/s13017-026-00714-x (PMC13679183; doi:10.1186/s13017-026-00714-x)
Supplement: Supplementary file 3 [file 13017_2026_714_MOESM3_ESM.docx]

**Appendix C: Respondent distribution**

**Table 4a** Independent response rates to each DSATC course questionnaire

| Course | Questionnaire | | | | | Total |
| --- | --- | --- | --- | --- | --- | --- |
|  | Pre-course | Post 1 day | Post 3 months | Post 1 year | Post 2 years |  |
| 2016 | 36 (65%) | 20 (36%) | 15 (27%) | 11 (20%) | 9 (16%) | 55 |
| 2017 | 37 (61%) | 38 (62%) | 27 (44%) | 18 (30%) | 13 (21%) | 61 |
| 2018 | 47 (53%) | 52 (58%) | 26 (29%) | 19 (21%) | Missing | 89 |
| 2019 | 51 (59%) | 20 (23%) | 24 (28%) | 10 (11%) | Missing | 87 |
|  |  |  |  |  |  |  |
| Traditional  DSATC | 171 (59%) | 130 (45%) | 92 (32%) | 58 (20%) | 22 (8%) | 292 |
|  |  |  |  |  |  |  |
| 2021 | 52 (37%) | 88 (63%) | 20 (14%) | 13 (9%) | 6 (4%) | 140 |
| 2022 | 25 (19%) | 35 (26%) | 13 (10%) | 2 (1%) | 11 (8%) | 134 |
| 2023 | 36 (26%) | 73 (52%) | 23 (16%) | 33 (23%) | N.A. | 141 |
| 2024 | 65 (48%) | 56 (41%) | 43 (32%) | N.A. | N.A. | 136 |
|  |  |  |  |  |  |  |
| Redesigned DSATC | 178 (32%) | 252 (46%) | 99 (18%) | 48 (9%) | 17 (3%) | 551 |
|  |  |  |  |  |  |  |
| Combined | 349 (41%) | 382 (45%) | 191 (23%) | 106 (13%) | 39 (5%) | 843 |

*(number)* percentage of total number of participants, *N.A.* not applicable since questionnaire has not yet been distributed to participants or collected
Percentages are rounded and may therefore not sum to 100.0%

**Table 4b** Longitudinal response rates to DSATC course questionnaires among ongoing participants

| Course | Questionnaire | | | | | Total |
| --- | --- | --- | --- | --- | --- | --- |
|  | Pre-course | Post 1 day | Post 3 months | Post 1 year | Post 2 years |  |
| 2016 | 36 (65%) | 17 (31%) | 10 (18%) | 5 (9%) | 2 (4%) | 55 |
| 2017 | 37 (61%) | 19 (31%) | 9 (15%) | 4 (7%) | 3 (5%) | 61 |
| 2018 | 47 (53%) | 28 (31%) | 17 (19%) | 9 (10%) | Missing | 89 |
| 2019 | 51 (59%) | 3 (3%) | 1 (1%) | 1 (1%) | Missing | 87 |
|  |  |  |  |  |  |  |
| Traditional  DSATC | 171 (59%) | 67 (23%) | 37 (13%) | 19 (7%) | 5 (2%) | 292 |
|  |  |  |  |  |  |  |
| 2021 | 52 (37%) | 40 (29%) | 12 (9%) | 4 (3%) | 3 (2%) | 140 |
| 2022 | 25 (19%) | 15 (11%) | 6 (4%) | 1 (1%) | 1 (1%) | 134 |
| 2023 | 36 (26%) | 25 (18%) | 13 (9%) | 7 (5%) | N.A. | 141 |
| 2024 | 65 (48%) | 33 (24%) | 17 (13%) | N.A. | N.A. | 136 |
|  |  |  |  |  |  |  |
| Redesigned DSATC | 178 (32%) | 113 (21%) | 48 (9%) | 12 (2%) | 4 (1%) | 551 |
|  |  |  |  |  |  |  |
| Combined | 349 (41%) | 180 (21%) | 85 (10%) | 31 (4%) | 9 (1%) | 843 |

*(number)* percentage of total number of participants, *N.A.* not applicable since questionnaire has not yet been distributed to participants or collected
Percentages are rounded and may therefore not sum to 100.0%
